# Diode Laser Turbinate Reduction in Allergic Rhinitis: A Cross-sectional Study

**DOI:** 10.31729/jnma.3870

**Published:** 2018-12-31

**Authors:** Priyanka Gupta, Toran KC, Deepak Regmi

**Affiliations:** 1 Department of ENT-Head and Neck Surgery, Bluecross Hospital, Kathmandu, Nepal; 2 Department of ENT-Head and Neck Surgery, Nepal Mediciti Hospital, Kathmandu, Nepal; 3 Department of ENT-Head and Neck Surgery, Kathmandu Medical College, Kathmandu, Nepal

**Keywords:** *allergic rhinitis*, *diode laser*, *inferior turbinate hypertrophy*, *turbinate reduction*

## Abstract

**Introduction:**

Turbinate reduction procedures are recommended for inferior turbinate hypertrophy in allergic rhinitis that fail to respond to medical therapy. Several modalities like turbinectomy, submucosal resection and tissue ablation are available for this purpose. The study aimed to evaluate the effectiveness of diode laser in the treatment of symptomatic inferior turbinate hypertrophy in allergic rhinitis and explore complications related to the procedure.

**Methods:**

This descriptive cross-sectional study was carried out in a tertiary care centre. The study enrolled 60 patients with inferior turbinate hypertrophy with failure of medical therapy. Inferior turbinate reduction was performed under local anaesthesia using diode laser. All the patients were evaluated subjectively for various nasal symptoms using visual analogue score scale preoperatively and during postoperative visit at three months.

**Results:**

The age ranged from 16 to 47 years with median age of 28 years. Twenty nine were male and thirty one were female. There was significant improvement in symptoms like nasal obstruction, nasal discharge, sneezing and decreased sense of smell. Immediate post-operative pain, crusting and persistent nasal discharge were observed as complications of the procedure. However, there was no incidence of mucosal oedema and synechiae formation in our study.

**Conclusions:**

Diode laser turbinate reduction procedure is safe, minimally invasive and effective in relieving the symptoms associated with inferior turbinate hypertrophy in allergic rhinitis resistant to medical therapy and can be performed on a day care basis under local anaesthesia.

## INTRODUCTION

Surgery in allergic rhinitis is reserved for patients who fail to improve with medical management and have inferior turbinate hypertrophy.^1,2^ Tissue ablation procedures, using diode lasers, carbon dioxide lasers, argon lasers, neodymium-yttrium aluminium garnet lasers, holmiumyttrium aluminium garnet lasers, potassium-titanyl-phosphate lasers and radiofrequency, have been widely used in developed countries. These laser types differ on emitted laser wavelength, output power, wave emission and mode of application.^3^ These parameters have impact on light-tissue interactions and resulting tissue effects in the form of ablation, coagulation and carbonization.^4^

Diode lasers of 940nm, 980nm, 470nm and 1940nm wavelength have been employed.^5–10^ Diode laser turbinate reduction (LTR) is performed as an office procedure under local anaesthesia. The operating time ranged from two to twelve minutes.^5,7,8,10,11^ Significant improvement was noted by three months time. The reported complications included intraoperative bleeding-postoperative blood mixed nasal secretions, pain, crusting and synechia.^6–8,10,11^

There are limited studies on diode LTR in Indian subcontinent.^6–8^ This study aimed to evaluate the efficacy of diode LTR in medical therapy resistant allergic rhinitis and encourage its use among care providers.

## METHODS

This was a descriptive cross-sectional study conducted in outpatient department of ENT-Head and Neck Surgery in Kathmandu Medical College Teaching Hospital (KMCTH). KMCTH was a tertiary care centre based in Kathmandu- Nepal. The study used convenient sampling technique. The sample size was calculated by using the following formula:


$$\begin{array}{l} \text{Sample Size calculation (n):}{\text{z}}^{2}\text{pq/}{\text{e}}^{2}\\ \text{=}{\left(1.645\right)}^{2}\times 0.10\left(1-0.10\right)/{\left(0.10\right)}^{2}\\ =24 \end{array}$$

where

z= confidence interval at 90%, 1.645,
p= prevalence, 10%,^12^
q= 1-p
e= margin of error, 10%.

The study involved sixty patients over one year period from November 2012 to November 2013. The patients of allergic rhinitis with failure of medical therapy for three consecutive months were selected for diode laser turbinate reduction (LTR) and were enrolled after informed consent. Patients with grossly deviated nasal septum- nasal polyps and history of previous nasal surgeries were excluded. Ethical clearance was obtained from ethical clearance committee of KMCTH.

Each of the nasal symptoms- including nasal obstruction- nasal secretions- sneezing- headache-decreased sense of smell and snoring- at presentation were assessed subjectively using visual analogue scale (VAS) and were scored along the scale of zero to ten. A score of zero represented no symptoms and a score of ten implied highly symptomatic. Similarly- a score of zero represented no obstruction whereas a score of ten implied complete obstruction. All the patients in the cohort underwent nasal endoscopy for baseline assessment and to rule out nasal polyposis and other relevant findings like deviated nasal septum. The patients underwent diode LTR as an office procedure with due precautions under local anaesthesia using 15% xylocaine surface spray followed by two percent xylocaine injection locally. Fox diode laser of 980 nm wavelength from A.R.C laser was used in contact mode with soft bending silica fibre of 600 /vm diameterat five watt power. Multiple linear applications, six to eight times- were made along the inferior turbinate as required over the duration of two to three minutes.

Patients were observed for half an hour for bleeding before discharge and were given acetyl salicylic acid for a day for pain relief and normal saline nasal solution for two weeks to prevent crust formation. The patients were assessed in three months follow up. During third month postoperative visit- the baseline symptoms were reassessed using VAS score. Postoperative morbidities like pain- blood mixed discharge- crusting- mucosal oedema and synechiae were also evaluated during postoperative visit with nasal endoscopy.

All the data were analysed using statistical package for social sciences 18 (SPSS 18). Both descriptive and inferential statistics were used for data analysis. In descriptive analysis- mean and standard deviation; and median and interquartile range were used. In inferential statistics, Wilcoxon Signed Rank test was used to study the efficacy of diode laser.

## RESULTS

**Figure 1.**
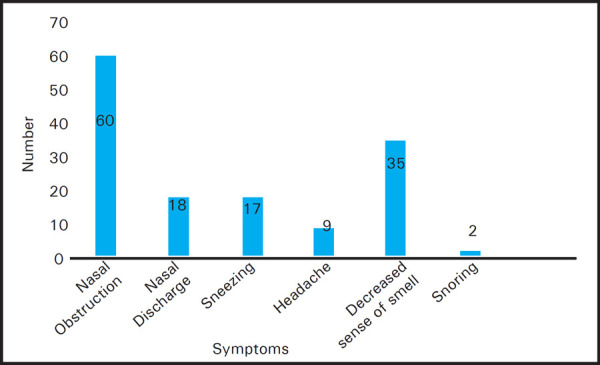
Frequency of symptoms at presentation.

The study enrolled 29 (48%) males and 31 (52%) females. The age group ranged from 16 years to 47 years. The median age in the study was 28 years. Nasal obstruction was the commonest symptom and was present in 100% (n=60) of the study population followed by decreased sense of smell 35 (58.33%), nasal discharge 18 (30%), sneezing 17 (28.33%), headache 9 (15%) and snoring 2 (3.33%) (Figure 1). The mean VAS score at presentation for nasal obstruction, decreased sense of smell, nasal discharge, sneezing, headache and snoring were 9.17, 3.78, 1.85, 2.07, 0.87 and 0.17 respectively (Table 1). The calculated P value for improvement of symptoms at three months was found significant for each of the symptoms except snoring. Each of the symptoms consistently decreased resulting in improvement of VAS score.

**Table 1:** Visual analogue scale (VAS) score at presentation and follow up visit.

Symptoms	VAS at presentation	VAS at three months
Nasal obstruction	9.17	1.75
Nasal discharge	1.85	0.27
sneezing	2.07	0.00
Headache	0.87	0.12
Snoring	0.17	0.05
Decreased sense of smell	3.78	1.30

The pain at laser site was consistently present in all the patients in the immediate post-operative period and was relieved uniformly in all the patients after sometime. Blood mixed discharge had similar course but was observed in only 22 (37%) patients. Nevertheless, crusting was noticed in 39 out of 60 (65%) patients at three months follow up. Mucosal oedema and synechiae were not observed in any patients during entire postoperative period (Table 2).

**Table 2:** Frequency of postoperative morbidities at three months follow up.

Complications	Immediate	Three month postoperatively
Pain	60	0
Blood mixed nasal discharge	22	9
Crusts	0	19
Synechiae	0	0
Mucosal oedema	0	0

## DISCUSSION

The study analysed the symptoms and postoperative morbidities with diode laser turbinate reduction (LTR) procedure in patients of allergic rhinitis refractory to at least three months of medical management. Inferior turbinate hypertrophy in allergic rhinitis causes nasal obstruction and hinders quality of living causing discomfort. Significant improvement was observed in each of the nasal symptoms by the end of three months. Nevertheless, unlike other symptoms, nasal obstruction and snoring had rather slower course for improvement. This was mainly due to post-operative swelling of coagulated tissue. The coagulated tissue sloughed off within four post-operative weeks and was eventually replaced by scar tissue. Postoperative oedema and crusting might be responsible for protracted improvement in nasal obstruction and sense of smell. There was decrease in nasal discharge by end of three months of laser therapy and could be attributed to destruction of highly vascular sub-mucosa and seromucinous glands.^8^ No patient reported sneezing at three months follow up. This could be due to destruction of branches of posterior nasal nerve.^8^

No significant complications were noticed during intraoperative period. All the patient complained of some degree of pain immediately following the procedure. The study would have been more informative if postoperative pain was also measured using a standardised scale. None of the patients however complained of pain in subsequent follow ups. Parida et al reported pain in only 30% of the patients in immediate post-operative period.^8^ Twenty two patients (37%) started having blood mixed nasal discharge following the procedure and nine patients (15%) had persistent blood mixed nasal discharge by the end of three months. Nineteen out of sixty (32%) had persistent crusting by the end of three months. The other studies with diode laser have not reported persistent nasal discharge at six months follow up.^6–8^ Hence, further assessment at six months follow up would have been valuable to assess nasal discharge in our study group. Mucosal oedema and synechiae formation were not observed in any patients postoperatively. The observation in the postoperative period suggested that the minimally invasive diode laser treatment was not associated with an increased risk of local infections unlike other surgical modalities.

## CONCLUSIONS

The study concluded that the diode laser turbinate reduction procedure, which could be performed as a day care office procedure, was effective in allergic rhinitis refractory to medical management. Almost all the patients experienced some pain despite analgesic measures prior to procedure. Stratification of pain in immediate post-operative would have made the study more informative. Persistence of crusts in some patients at three months follow up indicated the need of further follow up.

## Conflict of Interest

**None.**
